# Supplementary material for: Internationalization, cultural appreciation and institutional governmentality for quality control in transnational higher education cooperation: An empirical assessment
Source: PLoS One. 2022 Sep 22;17(9):e0274989. doi: 10.1371/journal.pone.0274989 (PMC9498945; doi:10.1371/journal.pone.0274989)
Supplement: Attachment — Submitted filename: Response to reviewers.docx [file pone.0274989.s001.docx]

**Dear Editor and honorable reviewers,**

**Thank you very much for your encouraging email to request a major revision of the paper coded as PONE-D-22-11647 “*Internationalization, cultural embeddedness and institutional governmentality for quality control in transnational higher education cooperation: An empirical assessment*”.**

**I am indeed grateful for your editorial decision to give us an opportunity to resubmit. I have closely examined the reviewers’ comments and your own suggestions, and then listed here the revisions I have made as follows with blue color bold font:**

Reviewer #1: The current study – “Internationalization, cultural embeddedness and institutional governmentality for quality control in transnational higher education cooperation: An empirical assessment” is very important to the development of TNE in China. While I commend the authors for the time and effort invested in the study, I think that there are additional work that need to be done in all the sections in order to bring the research to the journal’s standards. First, there is a lack of a clear theoretical underpinning of the study. I do not think that the authors’ discussions on internationalization and transnational higher education provide adequate information on the theoretical foundation. Secondly, although the introduction sets a very good pace for the delivery of very informative discussion on TNE and what the authors term as cultural embeddedness and institutional governmentality for quality control, the literature review and empirical sections were short of fulfilling those expectations. The major problem identified with the research findings is the lack of “thick data” or “participants’ narrative” to inform the findings. Summarily, although the current study is very important to the field of transnational higher education, I do not think that the authors have demonstrated enough evidence to show what new knowledge emerges from their study. I have provided some additional comments below for their attention. The Literature review part need to be revised. I think that the literature review section needs to be improved substantially to provide readers with better understanding of internationalization and transnational higher education. In its current form, the literature review section reads like an extension of the background section. The authors should provide answers to some vet important questions. For example: What are the theoretical foundations of internationalization and transnational higher education? What is already known in the field? What are the existing gaps in the concepts? Etc. unfortunately, these very important information cannot be found in the current text.

**Response to reviewer’s comments:**

**Thanks so much for your thoughtful feedback on this.**

**My heartfelt gratitude for the opportunity to come into contact with you and your wealth of knowledge.**

**The literature review section on pages 3-4 has been amended in response to your concerns, and it can be found in the revised article.**

**Geng and Zhao's conception of sustainable higher education and Tight's concept of globalization in discipline beyond the West have been cited in [29] [30] [31].**

**These citations add to the research on institutional globalization in higher education and the quality-based assurance for cooperation in higher education.**

Research site and research methodology
The authors have not provided adequate information concerning the population of their study, the sampling technique adopted to select participants for the study and how the questions were developed. I read the literature review section again to find out how the authors developed their research questions but I could not gather adequate information on that. Also information on the process used to analyze the data is very scanty. Seeing that that authors followed a systematic investigation process, readers should be informed about how the codes were developed into the three main themes. This further connects to my earlier comment concerning the gap between the questions and the theories/concepts used by the authors.

**Response to Reviewers: Your questions are really helpful to me as I work on revising the methods and spectrum section of the paper. A better explanation of research technique serves as a better explanatory chapter for a clearer research spectrum, so your questions are very important to the process.**

**Additionally, it is helpful in establishing a suitable methodological framework for the study results as well as the case studies part of the paper.**

**In this portion of the rewritten text, for example, I made significant changes to the research spectrum and research methodology on page 7, which includes a full discussion of the stakeholder approach.**

**In the portion of the approach that explained the process, I presented all of the stakeholders with the primary interview questions that were used in the article. This allowed for a more in-depth thematic analysis to be conducted in the section that detailed the results.**

**A more thorough explanation of the study process may serve as a better explanatory chapter for a more transparent research spectrum.**

Research findings
I struggled to identify how the narratives of the focus group participants were captured in the findings section. Did the researchers decide to abbreviate the contributions of participants? I suggest that the authors take a second look at the content and structure of the research findings section and provide readers with the rich text that reveal the voices of study participants. The discussion section also relies heavily on the secondary data analysed rather than the findings from the narratives of participants.

**Response to Reviewers: I really appreciate you taking the time to share your thoughts.**

**I have attended to your most insightful remarks in the same manner.**

**The research results here shown in the paper have shed light on the most significant issues raised by the participants.**

**To provide a final and summing-up account of the thoughts contributed by the interviewed participants is not the same thing as to minimize their involvement in the process.**

**The authors' goal is to reduce the length of the word count while maintaining a concise and accurate presentation of the material, which is why we have chosen to make use of summarized data. As a consequence of this, the manuscript does not include any direct citations that precisely quote what the interviewees stated word for word.**

**We really hope that such an approach will not be detrimental to the academic rigor and flavor of the piece that is being presented here.**

**Thank you very much for your support during this whole review. Your enthusiasm and contribution to this paper merit appreciation. After answering each of your review remarks individually, we began to celebrate the new appearance of the article we had created. Please accept our sincere appreciation, dear reviewer. Wish we could meet one day!**

Reviewer #2: The topic of this article is very relevant from the viewpoint of internationalization trends in higher education. The research methodology (PAR, case study) adopted in this study is proper. Conclusions are clear and well established. There are some typing mistakes (e.g. lowercase instead of capital letters) left in the text. Please correct them.

**Response to Reviewers:**

**Dear reviewer, thank you for your thoughts on the study on the issue of globalization tendencies in higher education!**

**According to your remarks we have made modifications appropriately.**

**We really appreciate your euthanasic remarks on the present study!**

**Thank you once again!**
